# Regucalcin Expression in Bovine Tissues and Its Regulation by Sex Steroid Hormones in Accessory Sex Glands

**DOI:** 10.1371/journal.pone.0113950

**Published:** 2014-11-21

**Authors:** Laura Starvaggi Cucuzza, Sara Divari, Chiara Mulasso, Bartolomeo Biolatti, Francesca T. Cannizzo

**Affiliations:** Department of Veterinary Sciences, University of Turin, Grugliasco, Torino, Italy; Hormel Institute, University of Minnesota, United States of America

## Abstract

Regucalcin (RGN) is a mammalian Ca^2+^-binding protein that plays an important role in intracellular Ca^2+^ homeostasis. Recently, RGN has been identified as a target gene for sex steroid hormones in the prostate glands and testis of rats and humans, but no studies have focused on RGN expression in bovine tissues. Thus, in the present study, we examined RGN mRNA and protein expression in the different tissues and organs of veal calves and beef cattle. Moreover, we investigated whether RGN expression is controlled through sex steroid hormones in bovine target tissues, namely the bulbo-urethral and prostate glands and the testis. Sex steroid hormones are still illegally used in bovine husbandry to increase muscle mass. The screening of the regulation and function of anabolic sex steroids via modified gene expression levels in various tissues represents a new approach for the detection of illicit drug treatments. Herein, we used quantitative PCR, western blot and immunohistochemistry analyses to demonstrate RGN mRNA and protein expression in bovine tissues. In addition, estrogen administration down-regulated RGN gene expression in the accessory sex glands of veal calves and beef cattle, while androgen treatment reduced RGN gene expression only in the testis. The confirmation of the regulation of RGN gene expression through sex steroid hormones might facilitate the potential detection of hormone abuse in bovine husbandry. Particularly, the specific response in the testis suggests that this tissue is ideal for the detection of illicit androgen administration in veal calves and beef cattle.

## Introduction

Regucalcin (RGN) was first identified in 1978 as a calcium (Ca^2+^)-binding protein [1], which does not contain the typical EF-hand Ca^2+^-binding motif [2]. Subsequently, RGN was identified as senescence marker protein-30 (SMP-30) based on the characteristic down-regulation of this protein with ageing in the rat liver [3]. As the name suggests, RGN regulates intracellular Ca^2+^ homeostasis through the modulation of the activity of Ca^2+^ channels, Ca^2+^-ATPase in the membrane of mitochondria and endoplasmic reticulum [4], [5] and (Ca^2+^-Mg^2+^)-ATPase in the plasma membrane [6], [7]. Moreover, RGN plays an important role in the regulation of Ca^2+^-dependent enzymes, such as protein kinases, tyrosine kinases, phosphatases, phosphodiesterase, nitric oxide synthase and proteases [8]–[13].

Several studies have showed a role for RGN in the regulation of cell death and proliferation; indeed, RGN also regulates DNA synthesis and fragmentation [14]–[17] and modulates the expression of oncogenes, tumour suppressor genes and cell cycle regulators [16]–[18], influencing cell survival and apoptosis [19]–[21].

RGN has been localised to the nucleus, cytoplasm [22]–[23] and the mitochondria [24]. RGN is widely expressed in a variety of tissues and cell lines [25]–[27] and was first identified in the liver, where this protein is highly expressed [1]. However, RGN mRNA and/or protein expression has also been detected in the male and female reproductive tract [23], [28]–[29], submandibular glands [30], several brain districts [13], [31]–[32], the heart [33]–[34], skeletal muscle [35], lung [36], kidney [35], [37], adrenal glands [19], bone [38]–[39]. RGN protein has been also shown to be secreted to biological fluids, namely plasma [31], [40]–[41] and seminiferous tubules fluid [29].

The expression of *RGN* is regulated through many factors, including intracellular Ca^2+^ concentration and regulatory transcription factors, namely transcription factor AP-1 [42], β-catenin [43], nuclear factor I-A1 (NF1-A1) [44] and *RGN* gene promoter region-related protein (RGPR-p117) [45]–[46]. In addition, Ca^2+^-independent mechanisms [47], including hormonal factors, such as thyroid, parathyroid and sex steroid hormones, have been described in the regulation of *RGN* expression in cells [23], [28], [48]–[49], [29]. The regulation of *RGN* expression through sex steroids in the rat liver [50], kidney [51] and more recently, the breast, prostate gland and testis [23], [29] has also been demonstrated.

Monitoring gene regulation via mRNA levels to detect anabolic sex steroid administration in bovine husbandry is a novel approach for the detection of the illicit treatment of livestock in meat production. The development of novel methods [52]–[56] to facilitate the indirect detection of the illegal administration of sex steroid hormones and other growth promoters would enhance the efficiency and success rate of food screening and safety programmes established by state authorities. Particularly, the transcriptomic approach could facilitate the identification of biomarkers suitable for the detection of illegally treated animals. Recent studies have shown that progesterone receptor (PR) gene expression levels were increased in the bulbo-urethral and prostate glands of 17β-estradiol-treated calves and beef cattle [53], [57]. For potential use in food safety monitoring, a quantitative PCR (qPCR) method has been developed for the detection of up-regulated PR gene expression in the bulbo-urethral glands of beef cattle and veal calves illegally administered 17β-estradiol [58].

Currently, there are no studies focusing on RGN expression in bovine tissues and organs. The first aim of the present study was to investigate RGN gene and protein expression in different tissues of veal calves and beef cattle. We also determined whether RGN expression is controlled through sex steroid hormones in bovine target tissues, namely the bulbo-urethral and prostate glands and the testis.

The significant change in *RGN* gene expression may well be an intriguing biomarker to discover hormone abuse in bovine husbandry. The described methodology, using an indirect marker to detect illegal hormone treatment, promises to significantly improve food safety control programs once introduced.

## Materials and Methods

### Animals

In trial 1, 18 Friesian male veal calves at 4 months of age were used. The calves were housed in 10×15 m boxes with concrete floors lacking litter or lateral partitions. The calves were tethered and fed with liquid milk replacer twice a day (providing per kg: 950 g dry matter (DM), 230 g crude protein (CP), 210 g ether extract (EE), 60 g ash, 1 g cellulose, 75 mg retinol, 50 mg ascorbic acid, 5 mg Cu, 0.125 mg cholecalciferol and 80 mg α-tocopherol). The amount of feed was gradually increased to 8 L/calf/day and then gradually increased to 16 L/calf/day. After one month, 0.5 kg of barley straw (per kg: 900 g DM, 20 g CP, 10 g EE, 60 g ash and 410 g crude fibre) was added to the diet, according to the recommendations of the European Commission (97/182/EC). The calves were randomly assigned to 3 experimental groups: group A (n = 6) received the weekly intramuscular administration of 17β-estradiol (dissolved in a preparation of ethyl oleate containing 10% of benzyl alcohol, added as bacteriostatic preservative) 6 times until 1 week before slaughter, for a total of 190 mg/animal; group B (n = 6) received the weekly intramuscular administration of testosterone propionate (diluted in sesame oil) 6 times until 1 week before slaughter, for a total of 1.050 g/animal; group K1 (n = 6) served as control. The first administration was carried out approximately at 140 days of age and the entire treatment phase lasted 44 days. The calves were slaughtered at 6 days after the last treatment.

In trial 2, 24 Friesian male beef cattle (7–15 months-old) were bought from local breeders and allowed to acclimatize for 2 months. The animals were housed in 10×15 m boxes with concrete floors lacking litter or lateral partitions. All animals were fed a concentrated diet comprising corn silage, corn, hay, and a commercial protein supplement; water was supplied *ad libitum*. The beef cattle were randomly assigned to 3 experimental groups at approximately 10–18 months-old: group C (n = 8) was administered 200 mg of trenbolone acetate and 20 mg of 17β-estradiol (Revalor-200, Intervet, USA) through slow-release subcutaneous pellets for 89 days; group D (n = 8) was administered 200 mg of trenbolone acetate (Finaplix-H, Intervet) through slow-release subcutaneous pellets for 89 days; group K2 (n = 8) served as control. The implants remained in place until slaughter.

The hormone dosages in both trials were selected according to previous studies [57], [59]–[62].

The animals in trials 1 and 2 were healthy upon *intra vitam* and *post mortem* examinations.

The experiments were authorised through the Italian Ministry of Health and the Ethical Committee of the University of Turin. The carcasses of the treated animals were appropriately destroyed (2003/74/CE – DL 16 March 2006, No. 158).

### Tissue sampling and processing

Parenchymal samples of the liver, kidney cortex, perirenal fat, adrenal cortex, adrenal medulla, lung, heart, skeletal muscle, cervical thymus, thoracic thymus, pituitary gland, salivary gland, bulbo-urethral gland, prostate and testis tissues were obtained from each animal. The sampling was carried out with the same procedure, immediately after the slaughter, for all animals. Samples were immediately frozen in liquid nitrogen and then stored at −80°C for molecular and western blot (WB) analyses. Samples from bulbo-urethral glands, prostate and testis of calves of trial 1 were also fixed in 10% neutral buffered formalin at room temperature and paraffin-embedded for immunohistochemistry (IHC).

### Chemicals

All chemicals, unless otherwise stated, were purchased from Sigma (St. Louis, MO, USA).

### RNA extraction, reverse transcription and PCR

RGN mRNA expression was analysed in the tissues of calves and beef cattle using polymerase chain reaction (PCR). Several milligrams of each tissue sample were disrupted using a TissueLyser II (Qiagen, Hilden, Germany) using stainless steel beads in 1 mL of TRIzol Reagent (Ambion, Life Technologies, Carlsbad, CA, USA) according to the manufacturer's protocol. The RNA concentration was spectrophotometrically determined, and the RNA integrity was evaluated using an automated electrophoresis station (Experion Instrument, Bio-Rad, Hercules, CA, USA). cDNA was synthesised from 1 µg of total RNA using the QuantiTect Reverse Transcription Kit (Qiagen).

The cDNA was subjected to PCR using Taq DNA Polymerase (Qiagen) and the following PCR protocol: initial denaturation (94°C for 3 min), followed by 35 amplification cycles (94°C for 1 min, 60°C for 1 min, 72°C for 1 min) and a final extension (72°C for 10 min). The primer sequences for RGN were designed using Primer3 software (vers. 4.0.0) based on reference sequence NM_173957. The size of the RGN amplicon (100 bp) was verified using an automated electrophoresis station (Experion Instrument, Bio-Rad), and DNA analysis was performed using the Experion DNA 1K Analysis Kit (Bio-Rad) [63].

### Quantitative expression analyses of RGN

The effect of sex steroid hormones on RGN mRNA expression in the bulbo-urethral glands, prostate and testis was evaluated through quantitative PCR (qPCR). To determine the relative amounts of specific RGN transcripts, the cDNA obtained from retrotranscription was subjected to qPCR [64] using the IQ5 detection system (Bio-Rad) and respective gene primers in an IQ SYBR Green Supermix (Bio-Rad). The primer sequences for RGN were the same as those used in the PCR assay. The cyclophilin A (PPIA) was used as a housekeeping gene, as previously described [57].

The levels of gene expression were calculated using a relative quantification assay based on the comparative Cq method (ΔΔCq method) [65], previously verifying that efficiencies of target and housekeeping gene amplification were similar. Subsequently, the relative abundance of each transcript, normalised to the endogenous housekeeping gene (PPIA) and relative to the control sample, was calculated as 2^−ΔΔCq^ (fold increase) [66]–[68].

### Western blotting analysis

RGN protein expression was analysed in the tissues of calves and beef cattle using WB. Total protein was isolated from bovine liver, kidney cortex, adrenal cortex, adrenal medulla, lung, heart, skeletal muscle, cervical thymus, thoracic thymus, salivary gland, bulbo-urethral gland, prostate and testis samples using RIPA buffer (50 mM Tris, pH 8.0, 150 mM NaCl, 1.0% IGEPAL CA-630, 0.5% sodium deoxycholate, 0.1% SDS and 2 mM EDTA) supplemented with protease inhibitor cocktail (Sigma). Total protein from perirenal fat and pituitary gland samples was isolated using TRIzol Reagent (Ambion, Life Technologies) according to the manufacturer's protocol. The protein concentration was determined using the Bio-Rad *DC* Protein Assay. Twenty micrograms of total protein were resolved through 12.5% SDS–PAGE. The proteins were blotted onto Trans-Blot TurboMini Nitrocellulose Transfer membrane (Bio-Rad) using a Trans-Blot Turbo Blotting System (Bio-Rad). The blotted membranes were blocked with 5% BSA in TBS-0.1% Tween for 1 h at room temperature, followed by overnight incubation with an anti-RGN rabbit polyclonal antibody (1∶200; Sigma). The membranes were subsequently incubated with secondary anti-rabbit horseradish peroxidase (HRP)-conjugated antibody (1∶1000), developed using the SuperSignal West Pico IgG Detection Kit (Thermo Fisher Scientific, Waltham, MA, USA) and recorded on CL-XPosure X-ray film (Thermo Fisher Scientific).

The effect of sex steroid hormones on RGN protein expression in the bulbo-urethral glands, prostate and testis of beef cattle was evaluated. In this case, α-tubulin (1∶10000, clone B-5-1-2; Sigma) was used as a total protein loading control.

### Immunohistochemistry

The bulbo-urethral glands, prostate and testis of veal calves were examined using IHC. The immunolocalisation of RGN was performed using an anti-RGN rabbit polyclonal antibody (Sigma). Briefly, the sections (4 µm) were deparaffinised and rehydrated. Endogenous peroxidase activity was blocked through incubation in 3% hydrogen peroxide for 15 min. After repeated rinsing with PBS, the sections were immersed in citrate buffer (10 mM, pH 6.0) and heated in a water bath at 98°C for 40 min for antigen retrieval. The sections were incubated with primary antibody at a 1∶100 dilution for 1 h at room temperature. The immunostaining was visualised using the EnVision Kit (Dako, Glostrup, Denmark) containing an HRP-labelled secondary antibody. Diaminobenzidine-hydrogen peroxide solution (Dako) was used as chromogen and applied for 5 min. The slides were subsequently rinsed in distilled water to terminate the reaction. After washing, the slides were counterstained with haematoxylin, dehydrated and mounted with a cover slip.

### Statistical analysis

Statistical analyses were performed using Graph-Pad InStat (vers. 3.05) statistical software (GraphPad Inc., San Diego, CA, USA). The analysis of RGN gene expression was performed using one-way analysis of variance (ANOVA), followed by Dunnett's post-test. If Bartlett's test suggested that the difference between the standard deviations of each group was significant, then the nonparametric Kruskal-Wallis test with Dunn's post-test versus the control group was applied. The Grubbs test was used to reveal potential outliers. A *P* value of <0.05 was considered statistically significant. The data are shown as the mean arbitrary units (2^−ΔCt^) ± SEM.

## Results

### RGN expression in various bovine tissues and organs

**Figure 1:**
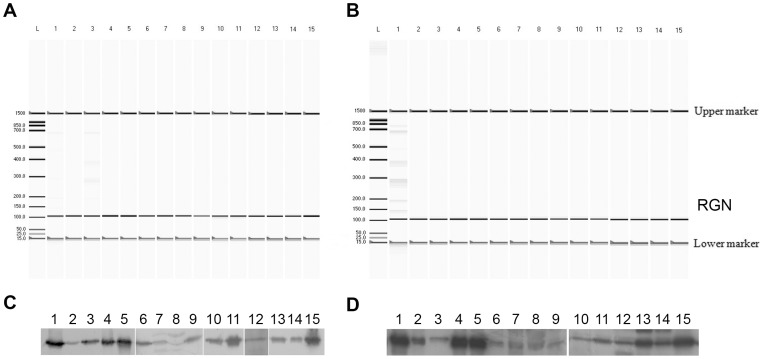
RGN expression in different bovine tissues and organs. A virtual gel of the automated capillary electrophoresis of the RGN amplicon (100 bp) in veal calves (A) and beef cattle (B). Two DNA internal markers (lower, 15 bp; higher, 1500 bp) were added to indicate peak alignments. A representative western blotting showing RGN protein expression in the different tissues and organs of veal calves (C) and beef cattle (D) using an anti-RGN rabbit polyclonal antibody (1∶200). Lane L: molecular weight marker; 1: liver; 2: kidney cortex; 3: perirenal fat; 4: adrenal cortex; 5: adrenal medulla; 6: lung; 7: heart; 8: skeletal muscle; 9: cervical thymus; 10: thoracic thymus; 11: pituitary gland; 12: salivary glands; 13: bulbo-urethral glands; 14: prostate; 15: testis.

The PCR analysis, using specific primers, demonstrated RGN mRNA expression in all tissues examined in both veal calves and beef cattle (Figure 1A and B). Following PCR, a single band of the correct length (100 bp) was detected. The presence of RGN protein in the same tissues was further confirmed through WB analysis (Figure 1C and D), showing a immunoreactive band of 33 kDa.

### Effect of sex steroid hormone on RGN expression in the accessory sex glands of veal calves

**Figure 2:**
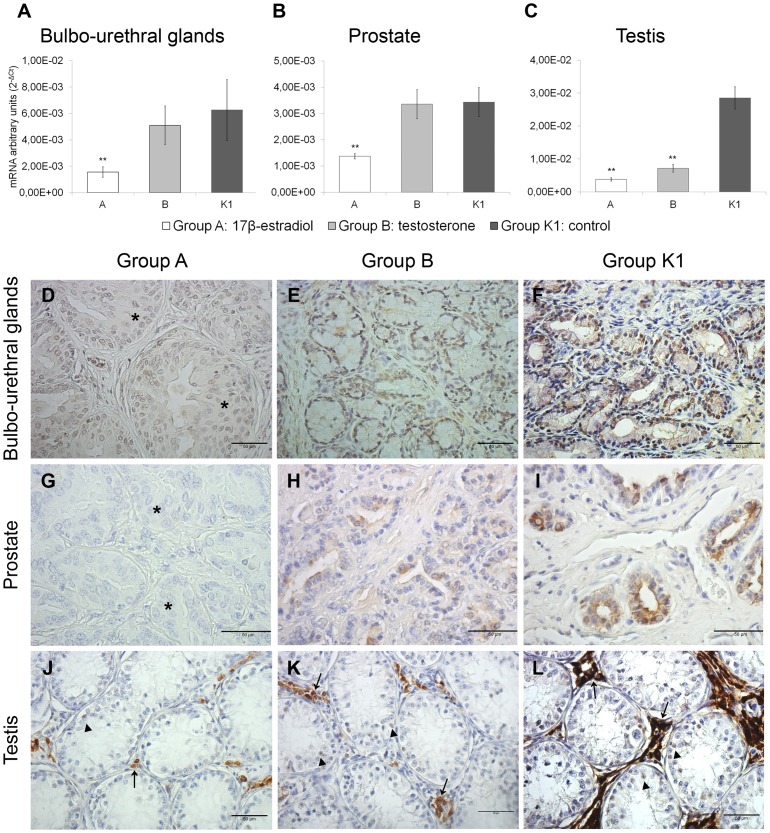
Effect of sex steroid hormones on RGN expression in the accessory sex glands and testis of veal calves. Effects of 17β-estradiol (group A) and testosterone (group B) on RGN gene expression compared with the control group K1 in the bulbo-urethral glands (A), prostate (B) and testis (C) of veal calves. The results are presented as the means ± SEM. The y-axes show arbitrary units representing relative mRNA expression levels. A representative image of the immunohistochemical localisation of RGN protein in paraffin sections of the accessory sex glands and testis of veal calves. RGN staining in the bulbo-urethral glands (D, E and F), prostate (G, H and I) and testis (J, K and L) of 17β-estradiol- (D, G and J), testosterone- (E, H and K) treated animals and control (F, I and L) animals. Typical hyperplasia and metaplastic lesions were observed in bulbo-urethral glands (D) and prostate (G) of 17β-estradiol-treated veal calves (asterisk). RGN protein was localised in the nuclei of glandular cells in the bulbo-urethral glands, in the cytoplasm of glandular epithelia in the prostate and in the cytoplasm of Leydig cells (arrows), although the nuclei of some spermatogonia (arrowheads) showed weak staining. Immunohistochemistry 400X. *P<0.05, **P<0.01 versus the control group K1.

In veal calves, 17β-estradiol administration (group A) decreased RGN expression in the bulbo-urethral glands (mean of mRNA arbitrary units ± SEM: 1.56E-03 ± 4.03E-04) compared with the control group K1 (6.26E-03 ± 2.30E-03) (*P*<0.01) (Figure 2A), in the prostate (1.37E-03 ± 9.86E-05) compared with the control group K1 (3.43E-03 ± 5.51E-04) (*P*<0.01) (Figure 2B) and in the testis (group A; 3.79E-03 ± 5.31E-04) compared with the control group K1 (2.85E-02 ± 3.35E-03) (*P*<0.01) (Figure 2C). Moreover, RGN expression was down-regulated in the testis of testosterone-treated veal calves (7.14E-03 ± 1.16E-03) compared with the control group K1 (2.85E-02 ± 3.35E-03) (*P*<0.01) (Figure 2C).

Immunohistochemical staining demonstrated RGN protein expression in the bulbo-urethral glands, prostate and testis of veal calves. RGN protein was localised in the nuclei of glandular cells in the bulbo-urethral glands (Figure 2D, E and F). The administration of 17β-estradiol (group A) revealed a strong decrease in RGN protein expression in this organ (Figure 2D) compared with the control group K1 (Figure 2F). Testosterone administration (group B) did not affect RGN protein expression in the bulbo-urethral glands (Figure 2E). RGN protein was localised to the cytoplasm of glandular epithelia in the prostate (Figure 2G, H and I). No staining was observed in the prostate of 17β-estradiol-treated calves (group A) (Figure 2G). Testosterone administration (group B) did not affect RGN protein expression in this organ (Figure 2H) compared with the control group K1 (Figure 2I). RGN staining in the testis was predominantly observed in the cytoplasm of Leydig cells, although the nuclei of some spermatogonia showed weak staining (Figure 2J, K and L). The immunohistochemical evaluation of the testis of calves treated with 17β-estradiol (group A) (Figure 2J) revealed a strong decrease in RGN protein expression compared with the control group K1 (Figure 2L). Only the testis showed a strong decrease in RGN expression following treatment with testosterone (group B) (Figure 2K) compared with the control group K1 (Figure 2L).

### Effect of sex steroid hormone on RGN expression in the accessory sex glands of beef cattle

**Figure 3:**
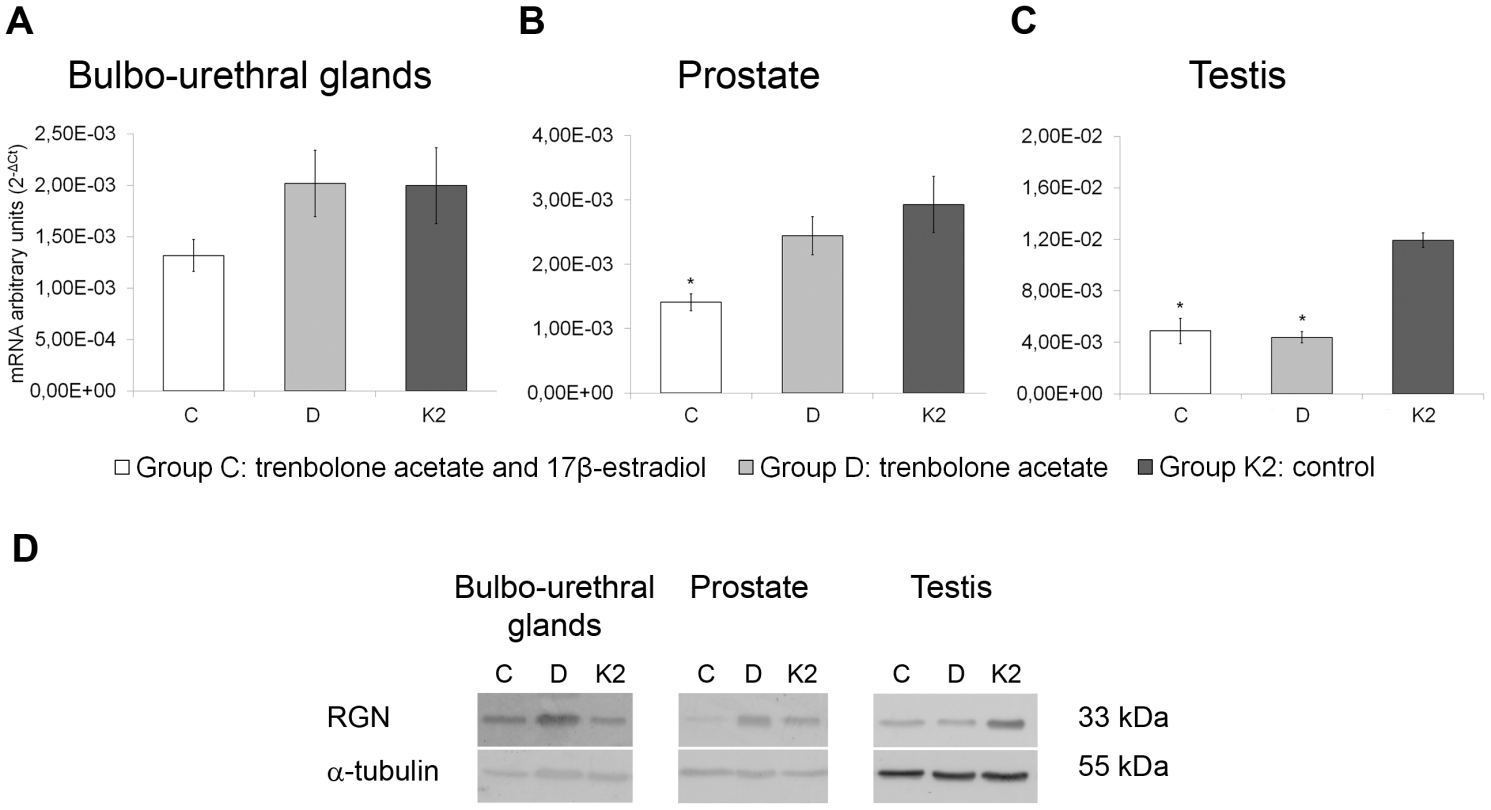
Effect of sex steroid hormone on RGN expression in the accessory sex glands and testis of beef cattle. Effects of trenbolone acetate and 17β-estradiol (group C) and trenbolone acetate alone (group D) on RGN gene expression compared with the control group K2 in the bulbo-urethral glands (A), prostate (B) and testis (C) of beef cattle. The results are presented as the means ± SEM. The y-axes show arbitrary units representing relative mRNA expression levels. A representative western blot (D) showing RGN protein expression in the bulbo-urethral glands, prostate and testis of beef cattle using an anti-RGN polyclonal antibody (1∶200). α-tubulin was used as loading control. *P<0.05 versus the control group K2.

Trenbolone acetate and 17β-estradiol administration (group C) in beef cattle reduced RGN expression in the prostate (1.41E-03 ± 1.32E-04) compared with the control group K2 (2.936E-03 ± 4.34E-04) (*P*<0.05) (Figure 3B) and in the testis (4.89E-03 ± 9.74E-04) compared with the control group K2 (1.19E-02 ± 5.68E-04) (*P*<0.05) (Figure 3C). Moreover, trenbolone acetate treatment (group D) down-regulated RGN expression in the testis (4.39E-03 ± 4.30E-04) compared with the control group K2 (1.19E-02 ± 5.68E-04) (*P*<0.05) (Figure 3C). No change in RGN expression was observed in the bulbo-urethral glands (Figure 3A).

The WB analyses demonstrated RGN expression in the bulbo-urethral glands, prostate and testis of beef cattle. No effect of sex steroid hormones was observed in the bulbo-urethral glands (Figure 3D). Treatment with trenbolone acetate and 17β-estradiol (group C) dramatically decreased RGN protein expression compared with the control group K2 in the testis and particularly in the prostate (Figure 3D). Moreover, treatment with trenbolone acetate alone (group D) down-regulated RGN protein expression in the testis compared with the control group (Figure 3D).

## Discussion

Although several studies have investigated RGN function in different species, the RGN expression in bovine tissues and organs has not been explored. To our knowledge, this study is the first to report RGN mRNA expression in bovine organs and tissues other than the liver [69].

WB analysis using an anti-RGN polyclonal antibody showed a reactive immunoprotein of approximately 33 kDa, corresponding to the predictive size of RGN. The IHC analyses also confirmed the RGN expression in the accessory sex glands, where the protein is localised to both the cytoplasm and nuclei of cells. Indeed, RGN translocates from the cytoplasm to the nucleus where this protein regulates DNA synthesis and fragmentation [14], [16]–[18], the expression of oncogenes, tumour suppressor genes and cell cycle regulators [17]–[18], [70]. Particularly, this cellular localisation pattern suggests a relevant role in testicular physiology in both veal calves and beef cattle. RGN is an important regulator of cellular Ca^2+^ homeostasis in several tissues. Ca^2+^ serves important biological functions, acting as a second messenger in several transduction pathways or regulating apoptotic cell death, among others [71]–[72]. A tightly regulated equilibrium between germ cell apoptosis and proliferation is required for a successful spermatogenesis [73], as approximately 75% of testicular germ cells undergo apoptosis [74]. Moreover, the tight control of intracellular Ca^2+^ homeostasis is critically important in the maintenance of Sertoli cell function [75]–[78] and Leydig cell steroidogenesis [79]–[80]. The histological evaluation of the testis from veal calves treated with 17β-estradiol or testosterone showed an interruption of germ cell line development, as previously described [57]. Particularly, testosterone administration caused a severe impairment with the reduction of germ cells to a layer of spermatogonia, with degenerated dark cells free in the lumen of seminiferous tubules [57]. This histological effect could be associated with the observed down-regulation of RGN gene expression in testis. Indeed, androgens are regulators of testicular cell death and considered as germ cell survival factors [81]–[84]. The *in silico* analysis of the RGN promoter region revealed different androgen response elements upstream from the transcription initiation site [28]. Moreover, the RGN-mediated regulation of apoptosis has been demonstrated *in vivo* and *in vitro* [15]–[19]. RGN inhibits apoptosis through the up-regulation of Akt-1 and Bcl-2 expression and the down-regulation of caspase-3 expression [85]. The anti-apoptotic effect of RGN has been demonstrated using knockout mice, whose cells are more prone to apoptosis than their wild-type counterparts [19], [86]. The reduction of RGN expression via androgen administration could inhibit normal spermatogenesis through the stimulation of abnormal apoptosis and the termination of germ cell line maturation.

RGN expression is modulated through estrogen hormones [23], [28]. The regulation of RGN expression through estrogens was first described in 1995 in the liver of rats receiving the subcutaneous administration of 17β-estradiol, resulting in an increase in RGN mRNA expression [50]. Conversely, the administration of 17β-estradiol reduced RGN expression in the kidney cortex of rats [51]. More recently, the effect of sex steroid hormones on RGN expression in the breast and prostate has been demonstrated [23], [28]. The administration of 17β-estradiol to rats induced the down-regulation of RGN expression in the prostate and mammary gland [23]. Consistent with these findings, we observed that the estrogen administration significantly decreased RGN expression not only in the prostate but also in the testis and bulbo-urethral glands. In particular, the estrogen administration caused a decrease of RGN expression in bulbo-urethral glands of veal calves, but not in beef cattle. This marked difference is likely due to physiological levels of the sex steroid hormones in adult male animals, as previously described for the expression of other estrogen-controlled genes [58]. Moreover, the different treatment schedule could influence the RGN expression.

It has been suggested that RGN has a physiological function in the prostate, as the expression of this protein is down-regulated in prostate cancer tissues, and RGN immunoreactivity is correlated with the grade of adenocarcinoma cellular differentiation [28]. Conversely, RGN expression and the estrogen-mediated down-regulation of this protein in the bulbo-urethral glands are reported for the first time in the present study. However, further studies are required to determine the precise RGN function in these organs. The effect of 17β-estradiol on morphology of the prostate and bulbo-urethral glands was confirmed by typical hyperplasia and metaplastic lesions observed in treated veal calves.

The effect of sex steroid hormones on *RGN* gene expression could play an important role in the indirect identification of animals illegally treated with hormones to improve the safety of meat production. This “omics” technology is based on the concept that after the identification of a specific transcriptional marker, it can be used to develop a novel screening method for the low-cost analysis of anabolic treatment in animal production. This approach has led to the identification of specific biomarkers for use in screening analyses to identify animals treated with sex steroid hormones. In recent years, for example, PR gene expression in the bulbo-urethral glands and prostate has been used as a biomarker for the illicit estrogen treatment of veal calves and beef cattle [53], [57]–[58]. Similarly, the variation of oxytocin gene expression in beef cattle muscle is indicative of estrogen and glucocorticoids illegal treatment [87].

In conclusion, we demonstrated the mRNA and protein expression of RGN in different bovine organs and tissues, demonstrating a pivotal multi-functional role for this protein in homeostasis regulation in tissues. In addition, the effect of sex steroid hormones on RGN expression in target organs, namely the bulbo-urethral and prostate glands and testis, suggests the potential detection of hormone abuse in bovine husbandry. Particularly, the specific response in the testis suggests RGN expression as the first molecular biomarker of illicit androgen administration in veal calves and beef cattle.
